# Cranial Shape Variation in Adult Kyrgyz Horses: Associations with Size and Sex

**DOI:** 10.3390/ani16203188

**Published:** 2026-10-11

**Authors:** Hasan Hüseyin Arı, Edyta Pasicka, Aycan Korkmazcan, Aybüke Akta, Ozan Gündemir

**Affiliations:** 1 Faculty of Veterinary Medicine, Kyrgyz-Turkish Manas University, Bishkek 720044, Kyrgyzstan; hasanh.ari@manas.edu.kg; 2 Department of Biostructure and Animal Physiology, Faculty of Veterinary Medicine, Wrocław University of Environmental and Life Sciences, Kożuchowska 1/3, 51-631 Wroclaw, Poland; 3 Institute of Graduate Studies, Istanbul University-Cerrahpasa, Istanbul 34320, Türkiye; aycan.korkmazcan@ogr.iuc.edu.tr (A.K.); aybuke.akta@ogr.iuc.edu.tr (A.A.); 4 Department of Anatomy, Faculty of Veterinary Medicine, Istanbul University-Cerrahpasa, Istanbul 34320, Türkiye

**Keywords:** Kyrgyz horse, cranial shape, geometric morphometrics, allometry, centroid size, sexual dimorphism, indigenous horses

## Abstract

Cranial diversity within indigenous horse populations remains incompletely characterized. Cranial shape variation and its associations with skull size and sex were investigated in 37 adult Kyrgyz horses aged 4–5 years (18 females and 19 males). Three-dimensional configurations of 25 cranial landmarks were evaluated using Procrustes alignment, principal component analysis, and permutation-based multivariate models. Skull size was quantified using centroid size calculated from the landmark configurations. The first two principal components represented 29.41% of shape variance, with changes distributed across multiple cranial regions and substantial overlap between sexes. A 5.42% greater mean centroid size was observed in males (*p* < 0.001). In separate models, 5.17% and 4.92% of shape variance were explained by sex and allometry, respectively (both *p* < 0.05). After size adjustment, neither an additional sex effect nor a size-by-sex interaction was statistically supported (both *p* > 0.05). Small, localized differences were observed between adjusted mean configurations. Cranial variation was therefore characterized by changes across several anatomical regions, a modest association with size, and limited evidence for additional sexual shape differentiation after size adjustment.

## Simple Summary

Skull shape variation in adult Kyrgyz horses was examined in relation to size and sex. Differences were observed across several skull regions, with considerable overlap between females and males. Larger skulls were generally found in males, but clear shape differences between the sexes were not detected after skull size was taken into account. Only a small proportion of shape variation was associated with size. These findings can be used as an anatomical reference for documenting indigenous horse diversity and interpreting skulls in teaching and museum collections.

## 1. Introduction

Cranial shape variation in domestic horses provides an opportunity to examine how anatomical diversity is expressed among breeds and among individuals within a population. The skull combines structures involved in feeding, sensory functions, and protection of the brain, making its morphology informative for comparative anatomy and the documentation of equine diversity. Studies of domestic horses and wild equids have demonstrated extensive cranial variation [1], while comparisons among horse breeds and landraces have linked cranial diversification to differences in size and breeding history [2]. Computed tomography combined with anatomical sections has also provided detailed descriptions of the Arabian horse skull and associated morphometric reference data [3]. Understanding variation within indigenous populations complements these investigations by establishing the anatomical range against which differences among populations can be interpreted.

Conventional morphometry has established useful descriptions of equine head conformation through linear measurements and indices [4]. However, selected lengths and widths provide only a partial representation of the spatial relationships among cranial structures. Digital approaches have also been applied to equine skeletal anatomy, with scanning and three-dimensional printing enabling the reproduction of identifiable anatomical features of thoracic limb bones [5]. For quantitative shape comparisons, three-dimensional geometric morphometrics examines the relative positions of corresponding anatomical points, termed landmarks, allowing differences to be quantified and visualized across the sampled configuration [6]. This approach distinguishes overall size from shape, but removing uniform differences in scale does not eliminate allometry, defined as the association between size and shape [7]. Consequently, skulls of different sizes may also differ in proportions, and apparent differences between groups require explicit evaluation of their relationship with size.

Previous geometric morphometric studies illustrate several sources of equine cranial variation. Comparisons of miniature, pony, and larger horse breeds have demonstrated differences in cranial growth trajectories, showing that adult proportions depend partly on the rate and duration of growth [8]. Analyses of horses from different age groups have likewise identified an allometric component to age-associated cranial differences [9]. At a more localized level, facial profile analyses have documented variation in nasal curvature across breeds and considerable diversity within Arabian horses [10]. Together, these findings show that cranial morphology varies at several biological scales. Patterns identified across breeds or developmental stages therefore require direct evaluation before they can be extended to variation among adults within a single population.

The Kyrgyz horse offers a relevant setting for examining such variation in an indigenous Central Asian population maintained under pasture-based management. Recent research has described its external conformation and age-associated differences in body and head measurements [11]. A subsequent study of adult Kyrgyz horse skulls using linear craniometry found pronounced differences in absolute size between females and males, whereas evidence for differences in size-corrected proportions was limited [12]. These observations provide a basis for investigating cranial form, but they do not establish how anatomical landmarks vary jointly in three dimensions or which spatial changes characterize the principal patterns of variation within this population.

The relationship between sex and cranial shape also warrants examination within this broader context. Sex-associated differences could reflect the distribution of females and males along a common relationship between size and shape, or could include differences that remain after size adjustment [7]. The latter possibility cannot be excluded from equine morphology in general: Hanot et al. detected differences between female and male cranial shapes after removing allometric effects in a comparative horse sample [2]. Their findings and the limited evidence for size-corrected differences in Kyrgyz horses [12] concern different samples and methods, underscoring the need for population-specific assessment. Three-dimensional analysis of cranial landmark configurations in Kyrgyz horses can help clarify the distribution of individual shape variation and its associations with size and sex.

The aim of the present study was to characterize cranial shape variation in adult Kyrgyz horses and evaluate its associations with cranial size and sex, with particular emphasis on whether sex-associated shape differences persist after accounting for allometry. By examining the spatial configuration of anatomical landmarks, the study was designed to complement existing linear osteometric evidence and address the limited understanding of how cranial shape variation is distributed within this indigenous horse population.

## 2. Materials and Methods

### 2.1. Study Design and Osteological Material

This study included 37 adult Kyrgyz horse skulls (Equus ferus caballus), comprising 18 females and 19 males, from the reference osteological collection of the Faculty of Veterinary Medicine, Kyrgyz-Turkish Manas University, Bishkek. The skulls originated from animals aged 4–5 years obtained from local slaughterhouses in Bishkek and surrounding areas of the Kyrgyz Republic. Sex was recorded at collection, and the skulls were prepared and maintained as dry osteological specimens. Complete configurations of 25 three-dimensional landmarks were available for all 37 specimens. The present investigation used existing collection material and involved no additional animal collection or procedures involving live animals.

### 2.2. Three-Dimensional Models and Landmark Acquisition

Three-dimensional surface models of the skulls were obtained using an EinScan Pro 2X structured-light scanner (SHINING 3D Tech. Co., Ltd., Hangzhou, China) operated in handheld mode. A turntable was not used because of the size of the specimens. Scan data were processed using the manufacturer’s EXScan Pro software (version 4.0.1.0), and the resulting polygonal surface models were exported in PLY format. The models were imported into 3D Slicer and inspected in multiple orientations to identify the anatomical structures required for landmark placement [13]. Landmark digitization was performed by a single observer (A.K.), following the anatomical definitions presented in Appendix A.

**Figure 1:**
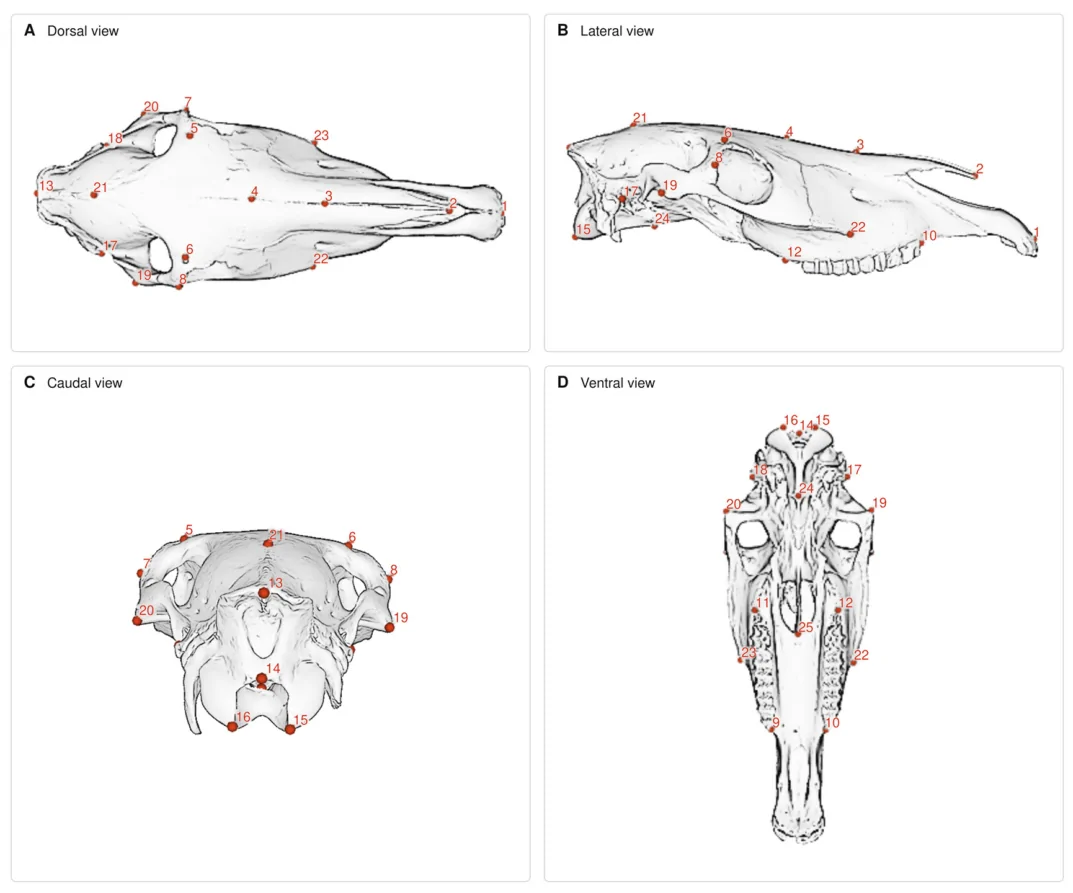
Cranial landmark configuration in (**A**) dorsal, (**B**) lateral, (**C**) caudal, and (**D**) ventral views. Red markers identify anatomical locations and numbers correspond to Table A1. Views are scaled independently for display; they do not provide a common physical scale.

Twenty-five fixed anatomical landmarks were recorded on each skull, representing median and bilateral structures in the rostral and nasal regions, cranial vault, orbital and zygomatic regions, maxillary and alveolar areas, basicranium, and occipital region. Curve and surface semilandmarks were not used. The spatial distribution and numbering of the landmarks are illustrated in Figure 1. Coordinates were exported as JSON markup files, with the same landmark sequence used for every specimen, and compiled into a dataset containing the specimen identifier, centroid size, and 75 coordinate variables.

### 2.3. Alignment and Data Quality Control

The original three-dimensional landmark coordinates from the first digitization were imported into R (version 2024.09.1) and organized as an array of 25 landmarks × 3 coordinate dimensions × 37 specimens. Landmark numbering and order were checked across specimens, and the dataset was examined for completeness and missing or non-finite coordinate values.

Generalized Procrustes analysis (GPA) was performed in R using the geomorph package to remove differences attributable to translation, rotation, and uniform scale [14,15,16]. Skull size was quantified using centroid size, calculated from the original coordinates before Procrustes scaling as the square root of the summed squared distances of the 25 cranial landmarks from their configuration centroid [7]. Thus, size in the present analyses refers specifically to the size of the cranial landmark configuration and does not represent overall body size. Centroid size was expressed in millimeters, whereas the GPA-aligned shape coordinates were dimensionless following scaling to unit centroid size. Following alignment, configurations were checked for centering at the origin and unit centroid size. Subsequent shape analyses used the GPA-aligned coordinates, whereas size analyses used the original centroid-size values. Because GPA does not remove size-associated shape variation, allometry was evaluated separately [7].

To assess intraobserver digitization repeatability, all 25 landmarks were placed twice on the same PLY surface model of each of the 37 skulls by a single observer (A.K.). Procrustes distances were calculated between the corresponding replicate configurations, and Procrustes ANOVA was used to evaluate differences between digitization sessions. The landmark configurations from the first digitization were used for all subsequent morphometric analyses, whereas the second digitization was used exclusively for the repeatability assessment.

### 2.4. Principal Patterns of Cranial Shape Variation

Principal component analysis (PCA) was performed in R (version 2024.09.1) on the GPA-aligned coordinates using the gm.prcomp function in geomorph (version 4.1.1) [15,16]. The first two principal components were plotted to describe the main axes of cranial shape variation, with specimens identified by sex and sex-specific convex hulls used to display their distribution. The percentage of total shape variance represented by each axis was reported. PCA was used for exploratory description; inferential tests were conducted on the full set of shape coordinates rather than on selected PC scores.

Shape configurations at the observed minimum and maximum scores of PC1 and PC2 were reconstructed from the mean configuration and the corresponding eigenvectors. These configurations were displayed in dorsal and lateral projections using an anatomical wireframe. For visualization, displacements from the mean configuration were magnified 1.8-fold. This magnification affected the displayed configurations only; statistical analyses and quantitative landmark displacements used the original values.

### 2.5. Cranial Size Analysis

Centroid size was summarized for females and males using the mean, standard deviation, standard error, median, interquartile range, range, and 95% confidence interval of the mean. Within-sex distributions were assessed with the Shapiro–Wilk test, and equality of variances was examined using the Brown–Forsythe procedure, calculated from absolute deviations around each sex-specific median. Female and male centroid sizes were compared using a two-sided Welch *t*-test, with a two-sided Wilcoxon–Mann–Whitney test used as a distribution-free sensitivity analysis. The magnitude of the difference was expressed as the male–female mean difference, its percentage relative to the female mean, and Hedges’ g, with positive values indicating larger male skulls.

### 2.6. Associations of Cranial Shape with Size and Sex

Associations between cranial shape, size, and sex were examined using Procrustes multivariate linear models in geomorph (version 4.1.1) and RRPP (version 2.2.0) [15,16,17]. Centroid size was natural-log transformed and centered by subtracting the sample mean, producing the predictor logCS_c. Overall static allometry was assessed using the model shape ~ logCS_c, and the unadjusted association between sex and shape was assessed using shape ~ sex [7].

The model shape ~ logCS_c + sex was fitted to evaluate the additional contribution of sex after accounting for size. Type I sums of squares were used, with logCS_c entered before sex. Potential differences in allometric relationships between females and males were evaluated using shape ~ logCS_c × sex, which included both main effects and their interaction. The size-only model was compared with the additive model to assess the incremental sex effect, and the additive model was compared with the interaction model to assess whether allowing sex-specific slopes improved model fit.

Models were fitted by ordinary least squares, and significance was evaluated using residual randomization under the reduced model [17]. Each test used 9999 randomized permutations in addition to the observed arrangement. Results were summarized using F statistics, explained proportions of shape variation (R^2^), standardized effect sizes (Z), and permutation *p* values. The randomization seed was set to 20260904.

Female and male mean shapes were calculated from the aligned configurations. Size-adjusted mean configurations were also predicted from the additive model at the overall mean log centroid size (logCS_c = 0). Their difference was expressed as the Euclidean distance between the predicted landmark configurations in the aligned coordinate space, and landmark displacement vectors were used to describe its anatomical distribution. For graphical presentation, displacements were magnified 1.8-fold around the midpoint of the two predicted configurations. These visualizations were interpreted alongside the full-shape tests. 

**Figure 4:**
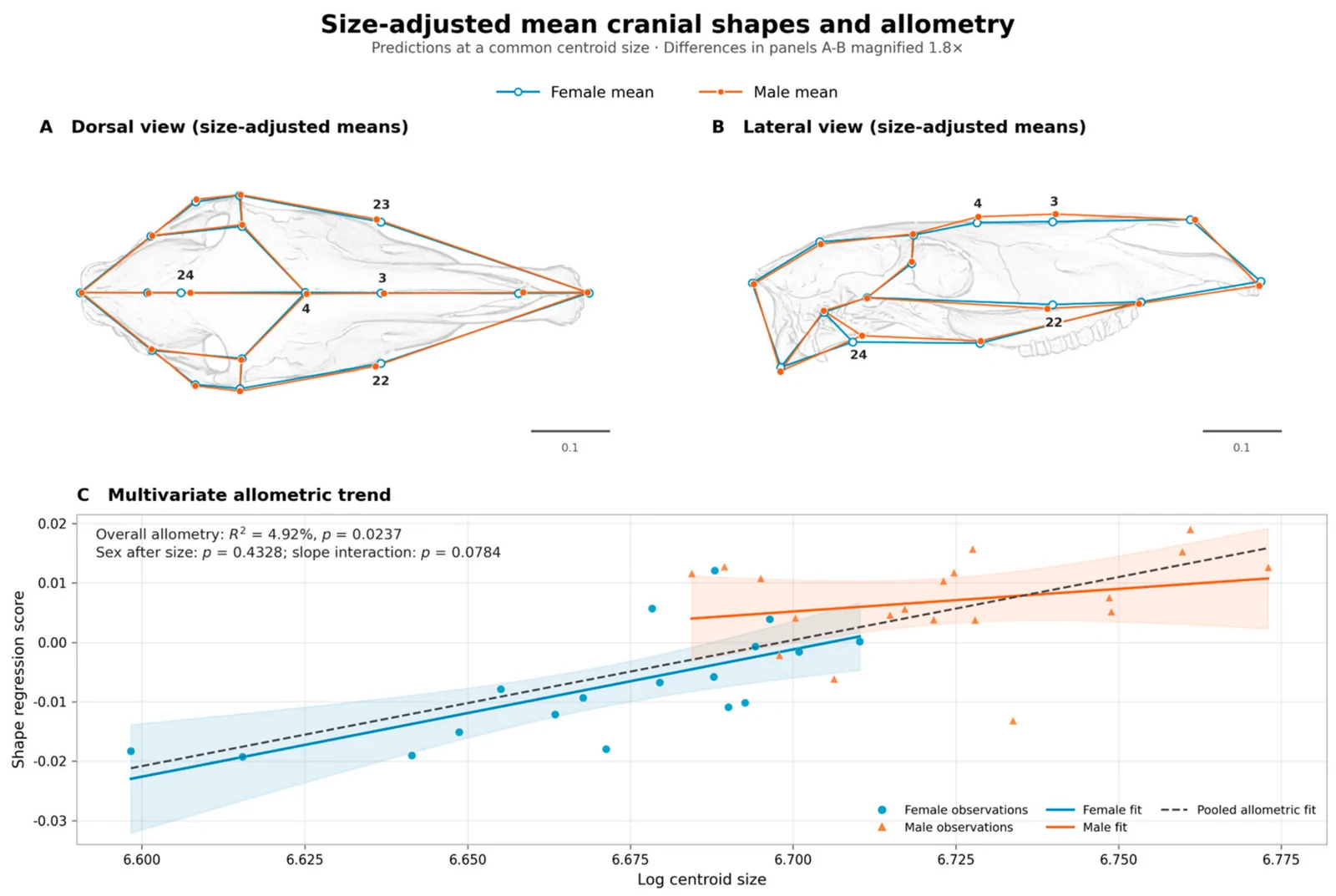
Size-adjusted mean cranial shapes and allometry in Kyrgyz horses. (**A**,**B**) Predicted female and male mean configurations in dorsal and lateral views, respectively. Blue open symbols and lines represent females, and orange filled symbols and lines represent males. Predictions were obtained from the additive shape model at the sample mean log centroid size and displayed using a common rigid three-dimensional rotation. Pale gray skull images provide static anatomical context, and numbers identify selected landmarks showing the largest adjusted mean differences. Connecting lines facilitate interpretation of cranial form. Female–male differences were magnified 1.8-fold around the midpoint of the two predicted configurations for visualization only; numerical results are based on unamplified shapes. Both panels use the same scale, with scale bars representing 0.1 dimensionless Procrustes coordinate units. (**C**) Shape regression scores plotted against log centroid size. Blue circles and orange triangles represent individual females and males, respectively. Solid lines indicate sex-specific linear fits, shaded bands represent 95% confidence intervals for the fitted mean scores, and the dashed gray line indicates the pooled allometric trend. Multivariate shape tests supported overall allometry (R^2^ = 4.92%, *p* = 0.0237), whereas neither the additional sex effect after size adjustment (*p* = 0.4328) nor the size-by-sex interaction (*p* = 0.0784) was statistically significant.

To visualize cranial allometry, shape regression scores were calculated by projecting the mean-centered Procrustes shape coordinates onto the unit-length regression coefficient vector estimated from the pooled model of shape on log centroid size. These scores were plotted against log centroid size for individual specimens. Sex-specific ordinary least-squares regression lines and 95% confidence intervals for the fitted mean scores were displayed together with the pooled regression line (Figure 4C). These plots provided a descriptive representation of allometric variation, while statistical inference was based on the multivariate shape models.

### 2.7. Statistical Software and Reporting

Statistical analyses were conducted in R version 4.5.2 [18], using geomorph version 4.1.1 [15,16], RRPP version 2.2.0 [17], and ggplot2 version 4.0.1. Statistical significance was evaluated at α = 0.05. Exact *p* values were reported where practicable, with values below 0.001 presented as *p* < 0.001.

## 3. Results

Procrustes ANOVA did not detect a statistically significant difference between the two landmark digitization sessions performed by the same observer (*p* > 0.05).

### 3.1. Sexual Dimorphism in Cranial Size

**Figure 2:**
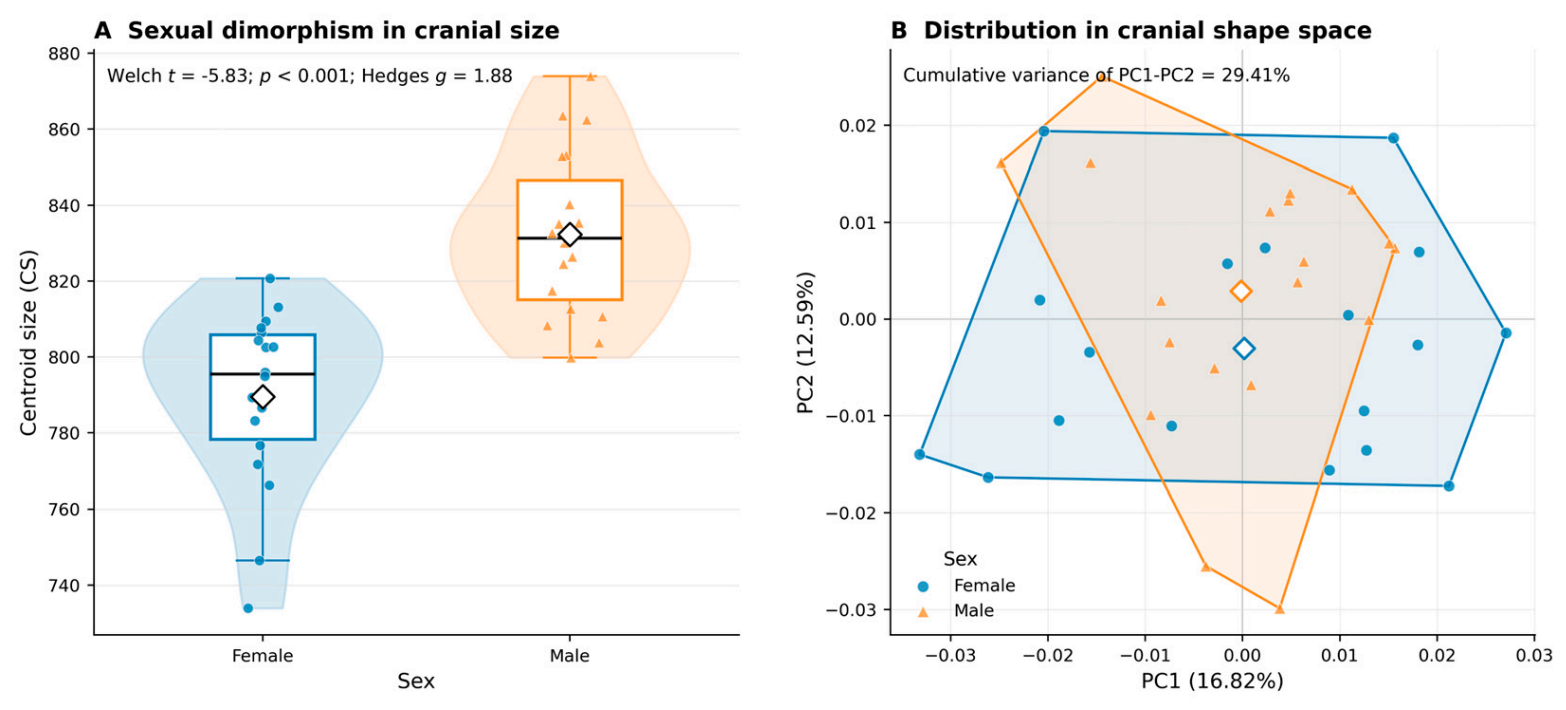
Sex-related variation in cranial size and overall shape space. (**A**) Distribution of centroid size in females and males. Points represent individual specimens, boxes show the median and interquartile range, and diamonds indicate group means. (**B**) PC1-PC2 ordination of GPA-aligned cranial shape coordinates. Lines and shaded areas indicate sex-specific convex hulls, while open diamonds indicate group centroids. PC1 and PC2 jointly explained 29.41% of total shape variance.

Centroid size was compatible with a normal distribution in both females (W = 0.914, *p* = 0.103) and males (W = 0.962, *p* = 0.608), and the Brown–Forsythe test did not detect a significant difference in variance between sexes (F(1,35) = 0.031, *p* = 0.861). Mean centroid size was 789.57 ± 23.27 mm in females and 832.36 ± 21.29 mm in males (Table 1). Welch’s independent-samples *t*-test identified a significant difference between the sexes (t(34.29) = −5.826, *p* < 0.001). The male–female mean difference was 42.79 mm (95% CI: 27.87–57.71 mm), corresponding to a 5.42% greater mean centroid size in males and a large standardized effect (Hedges g = 1.880). The Wilcoxon–Mann–Whitney sensitivity analysis supported the same conclusion (W = 22, *p* < 0.001; Figure 2A).

### 3.2. Cranial Shape Variation and the Unadjusted Sex Effect

The unadjusted Procrustes ANOVA detected a statistically significant sex effect on cranial shape (R^2^ = 0.0517, F = 1.907, Z = 2.112, *p* = 0.0173). Sex therefore accounted for approximately 5.17% of total Procrustes shape variance before centroid size was included in the model. In the principal component analysis, PC1 explained 16.82% of shape variance and PC2 explained 12.59%, for a cumulative contribution of 29.41%; the first four components together accounted for 50.41%. Female and male convex hulls overlapped extensively in the PC1-PC2 plane, and neither sex formed a discrete cluster on these two axes (Figure 2B). Because PC1 and PC2 represented less than one-third of total shape variance, this visual overlap does not conflict with the significant test based on the full multivariate Procrustes shape space.

### 3.3. Shape Changes Along PC1 and PC2

**Figure 3:**
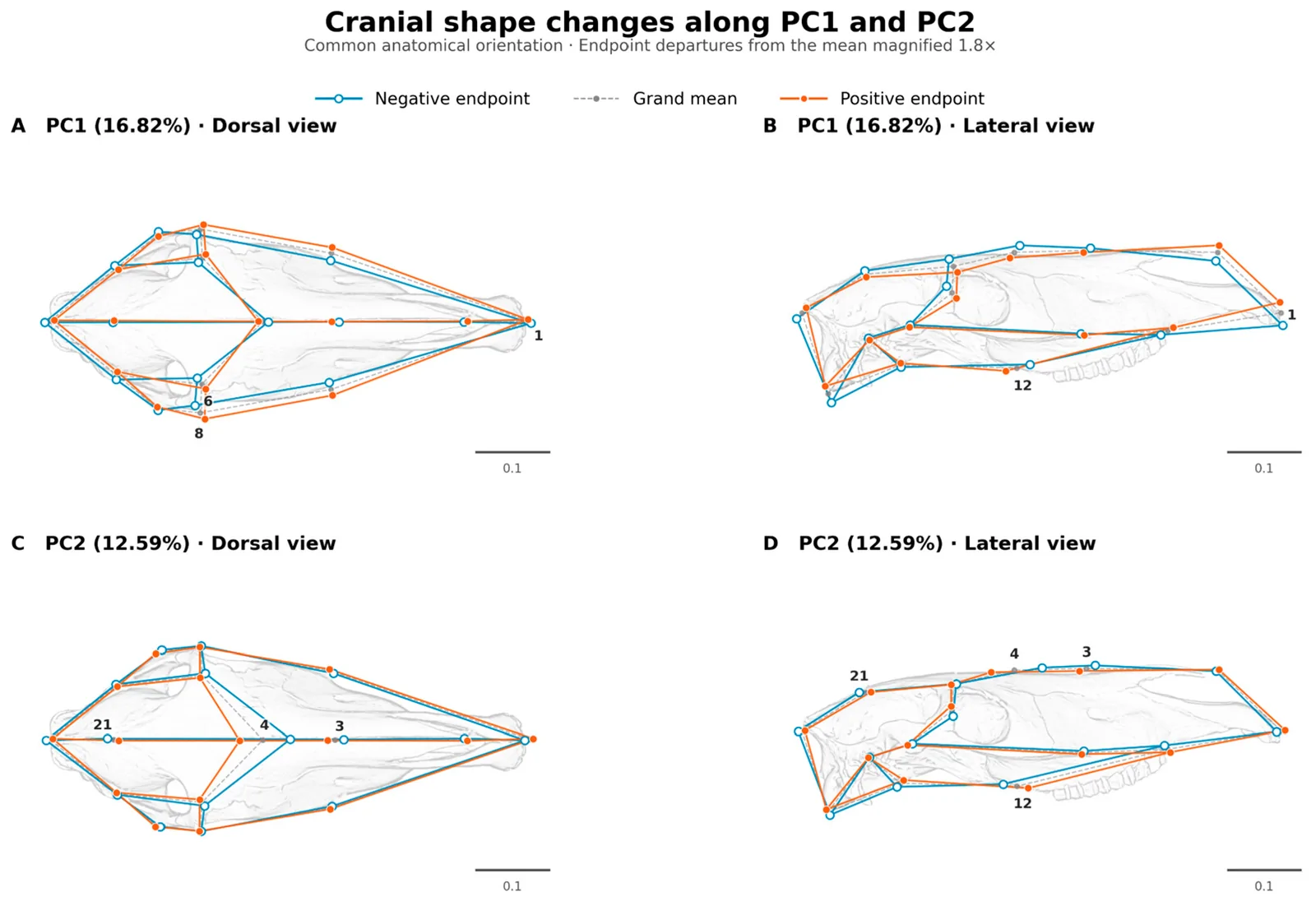
Cranial shape changes along PC1 (16.82%) and PC2 (12.59%) in anatomically oriented dorsal (**A**,**C**) and lateral (**B**,**D**) views of Kyrgyz horses. Blue open symbols and lines represent predicted configurations at the minimum observed scores of each principal component, whereas orange filled symbols and lines represent configurations at the maximum observed scores. The dashed gray wireframe indicates the grand mean. All configurations share a common rigid three-dimensional rotation. Pale gray skull images provide static anatomical context and are approximately aligned with the mean landmark configuration. Numbers identify selected landmarks illustrating the shape changes, and connecting lines facilitate interpretation of cranial form. For visualization, deviations of the endpoint configurations from the grand mean were magnified 1.8-fold; numerical results reported in the text remain based on unamplified configurations. All panels use the same coordinate scale. Scale bars represent 0.1 Procrustes coordinate units, which are dimensionless following centroid-size standardization.

Shape changes along PC1 and PC2 were visualized by comparing predicted configurations at the minimum and maximum observed scores in dorsal and lateral views (Figure 3). All endpoint displacement magnitudes reported below refer to the unamplified configurations and are expressed in dimensionless Procrustes coordinate units.

PC1 scores ranged from −0.0332 to 0.0271. The largest endpoint displacements occurred at the caudal alveolar margins of the left and right maxillary cheek-tooth rows (LM11 and LM12), measuring 0.0198 and 0.0196, respectively. Additional prominent displacements occurred at the rostral incisive point (LM1, 0.0180), the right caudolateral orbital rim (LM8, 0.0159), the right supraorbital foramen (LM6, 0.0147), and the rostral alveolar margin of the left maxillary cheek-tooth row (LM9, 0.0143). These changes involved the relative positions of landmarks in the incisive, maxillary alveolar, orbital, and supraorbital regions.

PC2 scores ranged from −0.0299 to 0.0251. The largest endpoint displacement occurred at the median frontonasal junction (LM4, 0.0390), followed by the caudal alveolar margins of the right and left maxillary cheek-tooth rows (LM12, 0.0200; LM11, 0.0173). Further changes involved the dorsal nasal point at the infraorbital level (LM3, 0.0130), the caudal frontal apex (LM21, 0.0090), and the right caudolateral orbital rim (LM8, 0.0080). This pattern described variation in the relative positions of dorsal nasal and frontal landmarks together with maxillary alveolar and orbital landmarks.

### 3.4. Size-Adjusted Mean Shapes and Allometry

Because centroid size differed significantly between the sexes, the comparison of mean shapes was additionally visualized after adjusting for size. Female and male predicted mean configurations were therefore estimated at a common centroid size using the model that included log centroid size and sex, and these size-adjusted means were displayed in dorsal and lateral views with an anatomically guided connecting network (Figure 4A,B). For readability, the visual separation between the two adjusted mean shapes in Figure 4A,B was amplified 1.8× around their common midpoint; the statistical results reported below are unaffected by this display choice.

The Euclidean distance between the size-adjusted female and male predicted mean configurations in aligned coordinate space was 0.01664. The largest localized adjusted mean-shape differences occurred at LM24 (0.0081 Procrustes shape-coordinate units), LM3 (0.0058), LM22 (0.0051), LM4 (0.0044), and LM23 (0.0042). These localized displacements remained visually modest, and their interpretation should be considered together with the formal test from the multivariate model, which did not support a statistically significant sex effect after size correction (*p* = 0.4328).

Across all specimens, cranial shape was significantly related to log-transformed centroid size (R^2^ = 0.0492, F = 1.813, Z = 1.966, *p* = 0.0237), indicating that size explained 4.92% of total Procrustes shape variance (Table 2; Figure 4C). After log centroid size was entered first in the model, the additional contribution of sex was not significant (incremental R^2^ = 0.0271, F = 0.998, Z = 0.164, *p* = 0.4328). The direct comparison of the size-only model with the size-plus-sex model yielded the same result (*p* = 0.4328). Therefore, the sex-associated shape component detected in the unadjusted model was not supported as an independent effect once cranial size was accounted for.

The log centroid size × sex interaction was also not statistically significant at the 0.05 level (incremental R^2^ = 0.0410, F = 1.532, Z = 1.430, *p* = 0.0784). Thus, the available data did not provide sufficient evidence that females and males followed different multivariate allometric slopes.

## 4. Discussion

The present study characterizes cranial shape variation within an adult Kyrgyz horse sample and shows that this variation extends across several regions of the landmark configuration. Cranial size was associated with a modest component of shape variation, while the contribution of sex depended on whether size was included in the model. These findings place sexual variation within the broader context of individual cranial diversity. They complement geometric morphometric studies of domestic and wild equids [1,2] by describing variation within one indigenous population and a restricted adult age range.

The first two principal components accounted for 29.41% of total shape variance, with PC1 explaining 16.82% and PC2 explaining 12.59%. Their combined contribution indicates that cranial variation was distributed across additional dimensions, so the two-dimensional ordination represents only part of the observed diversity. Female and male distributions overlapped extensively in this projection. Because PCA maximizes total variance without using sex labels, this overlap is a descriptive result and does not establish equivalence between the sexes [19]. It can therefore coexist with a significant sex effect in a test using the complete shape configuration.

The endpoint configurations help identify the anatomical distribution of the leading patterns of variation. The strongest PC1 displacements included LM11, LM12, LM1, LM8, LM6, and LM9, whereas PC2 emphasized LM4, LM12, LM11, LM3, LM21, and LM8. Thus, both axes involved changes in the relative positions of spatially separated landmarks. Comparative equid studies likewise describe variation involving facial proportions, skull breadth, the cranial vault, and the occipital region [1,2]. However, principal components depend on the specimens and landmarks included in an analysis. The Kyrgyz horse axes consequently cannot be treated as anatomical equivalents of similarly numbered axes in other studies.

These coordinated landmark displacements describe covariance within the sampled configuration, but the present analyses did not test cranial integration or modularity. Such questions require explicit hypotheses about anatomical subdivisions and quantitative evaluation of covariation within and between them [20]. Heck et al. [1] and Hanot et al. [2] addressed these questions using dedicated analyses. The present PCA provides a basis for selecting regions for subsequent investigation, without establishing that these regions constitute developmental or functional modules.

Mean centroid size was 5.42% greater in males, with a large standardized difference (Hedges’ g = 1.88). Arı et al. [12] similarly reported larger male means for all 18 linear cranial measurements, whereas no size-corrected variable or shape PCA score remained significant after false-discovery-rate adjustment. Conventional measurements have long provided useful descriptions of equine cranial proportions [4]. The present geometric morphometric analysis complements this literature by describing the spatial relationships among cranial landmarks and evaluating the association between sex and shape after adjustment for centroid size.

The change in the sex result after adjustment requires careful interpretation. Sex accounted for 5.17% of total shape variance in the unadjusted model, whereas its additional contribution after log centroid size was entered was 2.71% and was not statistically significant (*p* = 0.4328). Procrustes alignment removes uniform scale but retains associations between size and shape, making an explicit allometric analysis necessary [7]. The present results are consistent with some overlap between size-associated and sex-associated variation. However, the reduction in the sex term does not quantify a causal mediation effect, demonstrate that all sexual variation is attributable to size, or establish that the adjusted female and male population means are identical.

Overall allometry accounted for 4.92% of shape variance (*p* = 0.0237), leaving most variation unexplained by the size-only model. This relationship concerns static allometry among adults and should be distinguished from shape changes occurring during growth [7]. Heck et al. [8] reported that size explained 42% of cranial shape variation in 194 specimens representing 38 horse and pony breeds across developmental stages. Hanot et al. [2] also reported a strong size-shape association in a broader comparative sample, and Liuti and Dixon [9] found that removing allometric effects reduced the separation of horse age groups. The more restricted age and population composition of the present sample may contribute to its smaller allometric component. Differences in anatomical coverage and statistical procedures also preclude treating the reported percentages as directly comparable estimates of the same biological relationship.

Evidence for residual sexual shape variation differs among equine studies. Hanot et al. [2] detected significant female–male differences after removing allometric effects in a comparative sample encompassing several horse groups and substantially denser anatomical sampling. Differences in population composition, anatomical coverage, and analytical procedures complicate direct comparison with the present findings. Breeding history and environmental conditions could also contribute to variation among populations, but the contrast between these studies does not establish that Kyrgyz horses experience different sexual selection pressures. The present study did not compare selection regimes or directly evaluate adaptation. Such explanations therefore remain hypotheses requiring comparative evidence. Biologically, the significant difference in skull size shows that sexual dimorphism was detectable in this component of cranial form, even though an additional sex effect on shape was not statistically supported after adjustment for skull size.

Limited statistical power is another possible explanation for the non-significant adjusted sex effect. With 18 females and 19 males, the study may have had limited ability to detect subtle shape differences against individual variation. Sex accounted for an additional 2.71% of total shape variance after centroid size was included, but this effect was not statistically significant (*p* = 0.4328). This result neither establishes equivalence between the sexes nor demonstrates that insufficient power caused the non-significant finding. The configuration of 25 fixed landmarks also represents selected aspects of cranial morphology and may not capture differences elsewhere on the skull. The appropriate interpretation is therefore that, under the current sample and methods, no statistically significant sex-related cranial shape difference was detected after accounting for skull centroid size. Larger samples examined with comparable anatomical and analytical protocols are needed to assess whether subtle differences persist.

The skull size-by-sex interaction was not statistically significant (incremental R^2^ = 0.0410, *p* = 0.0784), but this result should not be interpreted as evidence that females and males have identical allometric slopes. With 18 females and 19 males, statistical power to detect subtle differences in the relationship between skull size and shape may have been limited. Consequently, a Type II error, whereby a genuine interaction remains undetected, cannot be ruled out. Nevertheless, the *p*-value alone cannot establish that insufficient power explains the result or confirm a sex-specific allometric trend. The common-slope model was therefore used as a parsimonious basis for visualizing adjusted mean shapes, without treating slope equality as demonstrated. Larger samples with adequate representation across the skull-size range within each sex are needed to estimate and compare these relationships more precisely. The separate fitted lines in the allometry plot describe projected shape regression scores and should be interpreted alongside the full multivariate interaction test.

The size-adjusted mean configurations showed small, localized differences involving the region between the muscular tubercles of the basilar part of the occipital bone, the dorsal midline of the nasal bones at the infraorbital level, the rostral ends of the facial crests, and the median frontonasal junction. These differences identify anatomical regions for further investigation, but do not establish a distinct sexual shape pattern, as the overall sex effect was not statistically significant after size adjustment. The 1.8-fold magnification used in the figures facilitates visualization and should not be interpreted as the original magnitude of these differences. Alhaddad et al. [10] likewise documented considerable variation in facial profiles among horse breeds and within Arabian horses, including three profile subgroups within the Arabian sample. However, their analysis concerned external facial profiles, so those subgroup patterns are not directly comparable with variation in the three-dimensional cranial configuration examined here.

The restricted age range reduces variation associated with large developmental differences, while limiting what can be inferred about cranial growth. Equine studies spanning developmental or adult age groups have demonstrated size-associated differences in cranial configuration [8,9]. External morphometry of Kyrgyz horses has also identified age-related differences in head length and several body dimensions [11]. Body mass and withers height were unavailable for the present skulls, so the relationship between cranial size and overall body size could not be evaluated. Likewise, these data do not allow the observed shape patterns to be attributed to nutrition, workload, breeding history, or adaptation to mountain environments.

Several sampling and measurement limitations define the scope of the findings. The sample comprised 37 adult skulls, limiting the extent to which the findings can be generalized to Kyrgyz horses across different regions and management systems. In addition, the 25 fixed landmarks describe the relative positions of selected anatomical structures but provide limited representation of the curves and surfaces between them. Future studies could extend this configuration using sliding curve and surface semilandmarks [21]. Along the dorsal nasal profile, additional curve sampling between LM2 and LM4 could characterize subtle differences in concavity, convexity, and the transition from the nasal to the frontal region that are incompletely represented by the existing points. Semilandmarks along the orbital margins could describe variation in orbital outline, including relative elongation, roundness, and localized curvature of the dorsal and ventral margins. Surface semilandmarks distributed over the nasal and frontal bones could additionally capture transverse curvature and localized flattening or prominence beyond the midline profile. These additions would permit more detailed assessment of whether sex- or skull-size-associated variation involves continuous contours and surfaces. Their contribution to statistical power would nevertheless require empirical evaluation. Future designs should define corresponding curves and surface patches consistently, document semilandmark density and sliding procedures, and assess digitization repeatability, while retaining comparable anatomical definitions across studies [6,21].

Repeated landmark digitization by the same observer enabled an assessment of intraobserver variation. However, the absence of a statistically significant difference between digitization sessions should not be interpreted as evidence that measurement error was absent. Random digitization error may still contribute to residual shape variance and influence the detection of small biological effects [22]. Because all landmarks were placed by a single observer, variation among observers was not evaluated. 

Further work should combine geographically broader sampling with individual body measurements and more detailed age information. Comparisons with Mongolian and other Central Asian horse populations would help determine whether the observed patterns extend beyond the current Kyrgyz series. Denser anatomical sampling and formal tests of integration could then examine how variation is distributed among cranial regions [20,21]. For collection documentation, the present configurations provide a reference for the sampled adults. Any subsequent application to individual sex estimation would require explicit assessment of predictive accuracy in independent material, since differences between group means do not establish classification performance.

Overall, the study extends the linear osteometric description of Kyrgyz horses by locating principal patterns of variation within a three-dimensional cranial configuration. Shape variation was distributed across multiple regions and was only modestly associated with centroid size. Male skulls were larger on average, while evidence for an additional sex effect on shape was limited after size adjustment. These findings provide a baseline for comparative work on indigenous horses and support evaluating cranial diversity through both individual variation and its associations with size and sex.

## 5. Conclusions

Cranial shape variation in adult Kyrgyz horses was distributed across several anatomical regions and was only modestly associated with skull size. Male skulls were larger on average, but under the current sample and methods, no statistically significant sex-related shape difference was detected after accounting for skull centroid size. This finding does not establish the absence of biological sexual dimorphism, and subtle shape differences may remain undetected. Evidence for differences in allometric slopes between the sexes was also insufficient. Existing linear osteometric evidence was extended by describing the spatial distribution of individual cranial variation, providing an anatomical reference for future comparisons among indigenous horse populations.

## Figures and Tables

**Table 1:** Descriptive statistics for centroid size (mm) according to sex.

Sex	n	Mean ± SD	Median (IQR)	Minimum-Maximum	95% CI
Female	18	789.57 ± 23.27	795.47 (27.57)	733.87–820.76	778.00–801.14
Male	19	832.36 ± 21.29	831.36 (31.47)	799.83–873.90	822.10–842.62

Note. SD, standard deviation; IQR, interquartile range; CI, confidence interval. Welch test: t (34.29) = −5.826, *p* < 0.001. Hedges g = 1.880 for the male–female contrast.

**Table 2:** Summary of Procrustes ANOVA and multivariate allometry models.

Effect Tested	Model	df	R^2^ (%)	F	Z	*p*
Unadjusted sex effect	Shape ~ sex	1, 35	5.17	1.907	2.112	0.0173
Overall allometry	Shape ~ logCS_c	1, 35	4.92	1.813	1.966	0.0237
Sex after size adjustment	Shape ~ logCS_c + sex	1, 34	2.71	0.998	0.164	0.4328
Size × sex interaction	Shape ~ logCS_c + sex + logCS_c:sex	1, 33	4.10	1.532	1.430	0.0784

Note. logCS_c denotes natural-log-transformed centroid size centered at the sample mean. Degrees of freedom are reported for the tested effect and residuals, respectively. R^2^ values represent percentages of total Procrustes shape variance. For sex after size adjustment, R^2^ indicates the incremental contribution of sex after logCS_c; for the interaction, it indicates the additional contribution of the logCS_c × sex interaction after both main effects. Z denotes the standardized effect size. Tests used 9999 residual randomizations in addition to the observed arrangement.

## Data Availability

The datasets generated during and analyzed during the current study are available from the corresponding author (O.G.) on reasonable request.

## Appendix A

Table A1 describes the 25 cranial landmarks illustrated in Figure 1. The numbering is retained from the original configuration and includes nine median landmarks and eight bilateral pairs. Anatomical terminology follows Nomina Anatomica Veterinaria [23].

**Table A1:** Anatomical locations of landmarks 1–25.

LM	Side	Anatomical Location and Description	View
LM1	Median	Rostral incisive point. Rostral end of the interincisive suture between the central upper incisors.	A, B
LM2	Median	Rostral nasal point. Rostral end of the internasal suture at the tip of the paired nasal bones.	A, B
LM3	Median	Dorsal nasal point at the infraorbital level. Point on the dorsal midline of the nasal bones corresponding to the dorsal projection of the midpoint between the centers of the right and left infraorbital foramina.	A, B
LM4	Median	Median frontonasal junction. Intersection of the frontonasal suture with the dorsal midline, at the junction between the frontal and nasal bones.	A, B
LM5	Left	Supraorbital foramen. Point at the dorsal opening of the left supraorbital foramen of the frontal bone.	A, C
LM6	Right	Supraorbital foramen. Point at the dorsal opening of the right supraorbital foramen of the frontal bone.	A, B, C
LM7	Left	Lateral orbital rim. Lateral prominence of the left caudolateral orbital rim in the region of the zygomatic process of the frontal bone.	A, C
LM8	Right	Lateral orbital rim. Lateral prominence of the right caudolateral orbital rim in the region of the zygomatic process of the frontal bone.	A, B, C
LM9	Left	Rostral maxillary alveolar margin. Rostral limit of the left maxillary cheek-tooth row, where the alveolar margin meets the diastema.	D
LM10	Right	Rostral maxillary alveolar margin. Rostral limit of the right maxillary cheek-tooth row, where the alveolar margin meets the diastema.	B, D
LM11	Left	Caudal maxillary alveolar margin. Caudal end of the left maxillary cheek-tooth row, at the alveolar margin adjacent to the last upper molar.	D
LM12	Right	Caudal maxillary alveolar margin. Caudal end of the right maxillary cheek-tooth row, at the alveolar margin adjacent to the last upper molar.	B, D
LM13	Median	External occipital protuberance. Median point on the external occipital protuberance, dorsal to the foramen magnum.	A, C
LM14	Median	Dorsal margin of the foramen magnum. Median point on the dorsal bony margin of the foramen magnum.	C, D
LM15	Right	Occipital condyle. Ventral extremity of the right occipital condyle, identified in caudal view.	B, C, D
LM16	Left	Occipital condyle. Ventral extremity of the left occipital condyle, identified in caudal view.	C, D
LM17	Right	Caudal margin of the external acoustic opening. Caudalmost point on the bony rim of the right external acoustic opening (porus acusticus externus).	A, B, D
LM18	Left	Caudal margin of the external acoustic opening. Caudalmost point on the bony rim of the left external acoustic opening (porus acusticus externus).	A, D
LM19	Right	Caudal zygomatic arch. Lateral prominence of the caudal part of the right zygomatic arch, adjacent to the mandibular articular region of the temporal bone.	A, B, C, D
LM20	Left	Caudal zygomatic arch. Lateral prominence of the caudal part of the left zygomatic arch, adjacent to the mandibular articular region of the temporal bone.	A, C, D
LM21	Median	Caudal frontal apex. Median point at the caudal apex of the V-shaped caudal margin of the frontal bones on the dorsal cranial surface.	A, B, C
LM22	Right	Rostral end of the facial crest. Rostral extremity of the right facial crest on the lateral surface of the maxilla, in the region of the facial tuberosity.	A, B, D
LM23	Left	Rostral end of the facial crest. Rostral extremity of the left facial crest on the lateral surface of the maxilla, in the region of the facial tuberosity.	A, D
LM24	Median	Median point between the muscular tubercles. Point on the ventral midline of the basilar part of the occipital bone, midway between the right and left muscular tubercles (tubercula muscularia).	B, D
LM25	Median	Caudal margin of the hard palate. Median point on the caudal border of the osseous hard palate, at the rostral boundary of the choanae.	D

## References

[1] Heck L., Wilson L.A.B., Evin A., Stange M., Sánchez-Villagra M.R. (2018). Shape variation and modularity of skull and teeth in domesticated horses and wild equids. Front. Zool..

[2] Hanot P., Bayarsaikhan J., Guintard C., Haruda A., Mijiddorj E., Schafberg R., Taylor W. (2021). Cranial shape diversification in horses: Variation and covariation patterns under the impact of artificial selection. BMC Ecol. Evol..

[3] Goodarzi N., Zehtabvar O., Tohidifar M. (2021). Applied anatomy of the skull in the Arabian horse: A computed tomographic, cross-sectional, volumetric and morphometric study. Vet. Med. Sci..

[4] Evans K.E., McGreevy P.D. (2006). Conformation of the equine skull: A morphometric study. Anat. Histol. Embryol..

[5] de Alcântara Leite dos Reis D., Gouveia B.L.R., Rosa Júnior J.C., de Assis Neto A.C. (2019). Comparative assessment of anatomical details of thoracic limb bones of a horse to that of models produced via scanning and 3D printing. 3D Print. Med..

[6] Szara T., Hadžiomerović N., Bakıcı C., Can Güzel B., Gündemir O. (2026). 3D geometric morphometrics in veterinary science: Applications, standardization, and future directions. Pol. J. Vet. Sci..

[7] Klingenberg C.P. (2016). Size, shape, and form: Concepts of allometry in geometric morphometrics. Dev. Genes Evol..

[8] Heck L., Sánchez-Villagra M.R., Stange M. (2019). Why the long face? Comparative shape analysis of miniature, pony, and other horse skulls reveals changes in ontogenetic growth. PeerJ.

[9] Liuti T., Dixon P.M. (2020). The use of the geometric morphometric method to illustrate shape difference in the skulls of different-aged horses. Vet. Res. Commun..

[10] Alhaddad H., Powell B.B., Del Pinto L., Sutter N., Brooks S.A., Alhajeri B.H. (2024). Geometric morphometrics of face profile across horse breeds and within Arabian horses. J. Equine Vet. Sci..

[11] Tasdelen S.M., Caglayan T., Salykov R. (2025). Morphometrical and morphological features of Kyrgyz native horses bred in Kyrgyzstan. J. Appl. Anim. Res..

[12] Arı H.H., Duro S., Alpak H., Tulobaev A., Ceylan N., Ashyk A., Salykov R., Djordjevic M., Gündemir O. (2026). Skull morphometry and sexual dimorphism in the Kyrgyz horse: A multivariate size-corrected analysis. Res. Vet. Sci..

[13] Fedorov A., Beichel R., Kalpathy-Cramer J., Finet J., Fillion-Robin J.-C., Pujol S., Bauer C., Jennings D., Fennessy F.M., Sonka M. (2012). 3D Slicer as an image computing platform for the Quantitative Imaging Network. Magn. Reson. Imaging.

[14] Rohlf F.J., Slice D. (1990). Extensions of the Procrustes method for the optimal superimposition of landmarks. Syst. Zool..

[15] Adams D.C., Otárola-Castillo E. (2013). geomorph: An R package for the collection and analysis of geometric morphometric shape data. Methods Ecol. Evol..

[16] Baken E.K., Collyer M.L., Kaliontzopoulou A., Adams D.C. (2021). geomorph v4.0 and gmShiny: Enhanced analytics and a new graphical interface for a comprehensive morphometric experience. Methods Ecol. Evol..

[17] Collyer M.L., Adams D.C. (2018). RRPP: An R package for fitting linear models to high-dimensional data using residual randomization. Methods Ecol. Evol..

[18] R Core Team (2025). R: A Language and Environment for Statistical Computing.

[19] Jolliffe I.T., Cadima J. (2016). Principal component analysis: A review and recent developments. Philos. Trans. R. Soc. A Math. Phys. Eng. Sci..

[20] Klingenberg C.P. (2009). Morphometric integration and modularity in configurations of landmarks: Tools for evaluating a priori hypotheses. Evol. Dev..

[21] Gunz P., Mitteroecker P. (2013). Semilandmarks: A method for quantifying curves and surfaces. Hystrix Ital. J. Mammal..

[22] Fruciano C. (2016). Measurement error in geometric morphometrics. Dev. Genes Evol..

[23] International Committee on Veterinary Gross Anatomical Nomenclature (2017). Nomina Anatomica Veterinaria.

